# Radiation oncology as part of medical education—current status and possible digital future prospects

**DOI:** 10.1007/s00066-020-01712-x

**Published:** 2020-11-23

**Authors:** Hendrik Dapper, Marjo Wijnen-Meijer, Salome Rathfelder, Katharina Mosene, Isabelle von Kirchbauer, Denise Bernhardt, Pascal O. Berberat, Stephanie E. Combs

**Affiliations:** 1 Department of Radiation Oncology, Klinikum rechts der Isar, Technical University Munich, Ismaninger Str. 22, 81675 Munich, Germany; 2 School of Medicine, TUM Medical Education Center, Technical University Munich, Nigerstraße 3, 81675 Munich, Germany; 3 DRF Stiftung Luftrettung gAG, Rita-Maiburg-Straße 2, 70794 Filderstadt, Germany; 4 Institute for Radiation Medicine (IRM), Helmholtz Zentrum München (HMGU), Ingolstädter Landstr. 1, Neuherberg, Germany; 5 Partner Site Munich, Deutsches Konsortium für Translationale Krebsforschung (DKTK), Munich, Germany

**Keywords:** Radiation oncology, Education, E‑learning, Flipped classroom, Seminars

## Abstract

**Purpose:**

Education as part of medical education is currently changing rapidly. Not least because of the corona crisis, more and more digital teaching formats and innovative teaching concepts such as the flipped classroom model are finding their way into teaching. We analyzed the acceptance and effectiveness of traditional teaching methods as well as the interest in innovative e‑learning methods among medical students in the field of radiation oncology at the medical school of the Technical University of Munich.

**Methods:**

We carried out an online-based survey as well as a knowledge test on all students from two terms who had completed the seminar series of radiation oncology. The survey comprised seven questions on the frequency of participation, acceptance, and judgment of the effectiveness in terms of learning and on a potential use of e‑learning methods using a six-point Likert scale. The test consisted of 10 multiple-choice questions.

**Results:**

Traditional teaching methods are largely accepted by students and most students consider the current learning format to be effective in terms of the teaching effect in the field of radiation oncology. However, only about 50% of all knowledge questions were answered correctly. The possible use of e‑learning methods was judged critically or desired in roughly equal parts among the students.

**Conclusion:**

Traditional seminars enjoy a high level of acceptance among students. Effectiveness with regard to the internalization of content taught, however, should be increased. After all, the future seems to lie in the integration of e‑learning in the form of educational videos and practical seminars.

## Background

Like many other fields of study, undergraduate medical education in Germany has undergone extensive changes in the past years. Although various innovative practice-oriented and case-oriented alternative types of education exist, the vast majority of medical students find themselves in the traditional one-dimensional teaching format for most of their time [1–3]. The Ohio State University College of Medicine conducted a survey and identified the most important components of medical education: next to clinical problem solving, learning how to acquire knowledge, developing bedside manner, teamwork, technology training, and clinical research were among the top six aspects [4]. All of these aspects require a high degree of reflection, empathy, teamwork, and practical skills, which must all be based on profound knowledge. While classroom teaching (with an expert lecturing and presenting information and students acting as passive listeners) has been the method of teaching for decades, innovative courses are replacing this traditional teaching method more and more and give students a more active role in an interactive setting. They are very well accepted among students [5]. A restructuring of the medical curriculum is therefore called for by education experts [6].

Introduction of new teaching methods in medical schools is driven to a large extent by digitalization. More and more digital formats find their way into medical education and are used by most students [7, 8]. One of the promising e‑learning formats is the “flipped classroom” [9]. According to this format, learning is divided into two steps. Firstly, students perform some pre-class activities [10]. For example, students watch an instructional video on their own, whenever and wherever they like. They learn the essential content of the video by themselves. In a second phase, students apply the knowledge acquired in interactive small group sessions facilitated by an expert [11]. In Germany, medical education remains very traditional, which is particularly true for the curriculum and the prerequisites, due to the central management of teaching and learning content as well as nationwide assessment and state examinations.

The COVID-19 crisis has not only pushed digital health but also led to social distancing in hospitals, to protect patients, medical workers, and students. As a consequence, face-to-face teaching was abandoned at many universities. The crisis thus promoted the necessity of employing digital teaching formats and, as a result, revealed their benefits [12, 13].

Besides surgery and clinical oncology, radiation oncology is one of the three main pillars in oncological therapy and is used for almost every tumor entity, depending on the stage of the tumor and the intention of tumor therapy [1, 14]. Due to its interdisciplinary importance and the fact that around two thirds of all tumor patients receive radiation therapy in the course of their disease, basic knowledge of indications and practical and technical implementation, as well as of clinically relevant aspects such as side effects and supportive therapies, should be taught to prospective physicians regardless of their final specialization [1, 15, 16].

At our medical school, radiation oncology seminars have so far been performed in the traditional form of face-to-face teaching. The teacher generally plays an active role, while the students mostly just absorb the information given and only start springing into action when they ask or are asked questions. In comparison to traditional lectures, however, there is at least a practical aspect to seminars, as they are closer to everyday clinical practice and mostly have a more intensive character due to the small groups. Therefore, seminars are generally very popular among students [17].

The aim of the study described in this article was to analyze the acceptance and effectiveness of traditional teaching methods in seminars, in this case radiation oncology education. Furthermore, we tried to find out to what extent students accept innovative e‑learning methods.

## Methods

The curriculum for medical education in Germany currently consists of four preclinical and six clinical semesters followed by two practical semesters. In accordance with the clinical curriculum of the medical school of the Technical University of Munich, radiation oncology (lectures and seminars) is currently taught together with radiology and nuclear medicine in a cross-sectional course called “imaging procedures, radiation therapy, and radiation protection.” It takes place in the first or second clinical semester and also in the third year of medical school. As about 160 students per semester have to take this course, but since for the seminars a group of 16 students is the maximum, each session has to be scheduled ten times per semester. With regard to radiation oncology, the course includes five different seminars of 45 min each. These seminars are compulsory, and every student has to attend them with the allowance of missing one session. The topics of these seminars are radiation biology, oncological informed consent discussion, radiotherapy process and linear accelerator, treatment planning, and brachytherapy. The main content of these seminars is summarized in Table 1.

**Table 1:** Seminar series “Radiation Oncology” at the Technical University of Munich

	Subject of the seminar	Main content; 45 min each	Implementation
1	Radiation biology	Definition of energy dose and radiation effects in cells	PowerPoint lecture given by radiation biologist
		Four R’s of radiotherapy, therapeutic breadth	
		Time scale of radiation effects in normal and tumor cells	Tour of the laboratory of radiation biology
		Assays to measure radiation effects	
2	Oncological informed consent discussion	Basics in conducting sensitive oncological conversations	Lecture given by a radiation oncologist
		Procedure and implementation of clarification talks	
		Indication, effects, and side effects of radiation therapy	Presentation and discussion of an information sheet for patients
		Legal aspects of clarification talks	
3	Radiation therapy process and linear accelerator	Implementation of the planning CT and patient positioning	Lecture given by a radiation oncologist
		Target volume definitions and consideration of organs at risk	
		Structure and functionality of linear accelerators	Brief demonstration of the linear accelerator and an irradiation session
		The process of a radiation therapy session, image guidance	
4	Treatment planning	Physical basics and properties of different therapeutic radiation	PowerPoint lecture given by a radiation physicist
		Important physical parameters in radiation therapy	
		Presentation of radiation plans and dose–volume histograms	Presentation of radiation plans and DVHs using radiation software
		Irradiation techniques (3D-RT, IMRT, IGRT)	
5	Brachytherapy	Different emitters and physical properties (LDR, HDR)	PowerPoint lecture given by a radiation oncologist
		Different methods of application of brachytherapy	
		Indication, planning, and implementation of brachytherapy	Tour of the premises and demonstration of brachytherapy equipment
		Special aspects of radiation protection	

### Procedure for and implementation of the five seminars

The course of the five seminars is structured as follows: from the students’ perspective, all five seminars take place on a weekly basis with the succession of the seminars depending on the student group. Each seminar unit is held in a small group of up to 16 students by an expert in the respective field, and takes a total of 45 min. From the teachers’ perspective, two teachers divide up their ten times of teaching the same subject. All five seminars are facilitated by the respective expert by means of a lecture. This lecture normally takes 20 to 25 min. Afterwards, equipment, premises, software applications, information sheets, or processes are also introduced to the students in 20 to 25 min, depending on the type of seminar (Table 1). It depends on the teacher to what extent students may get actively involved in class during lectures, presentations, and discussions arising from questions asked by students. These questions usually may be asked either during or at the end of each seminar. It is the teachers’ task to check the students’ presence and to sign a routing slip at each seminar. In addition, each student receives a handout of one to two pages (size DIN A4) at the end of each seminar which summarizes the main content of the lecture in key points and by means of graphics.

### Survey

For our survey we made use of Evasys, which is a chargeable web-based survey tool invented by the Electric Paper Evaluationssysteme GmbH (Lüneburg, Germany) [18]. This tool provides an automated evaluation of the number of participants, mean and median values, standard deviation, most frequent answers, and percentage of the respective answers. These results are displayed in both absolute numbers with one decimal place and in graphics. A total of 17 questions were drawn up. Only one out of five (knowledge test) and six (opinion) answers, respectively, was to be chosen.

The survey was divided into two sections.

- The first section related to the personal opinion of the students and comprised seven questions on the frequency of participation, acceptance, and judgment of the effectiveness in terms of learning, and on a potential use of e‑learning methods in connection with the radiation oncology seminars. In most cases, students were supposed to choose one answer on a six-point Likert scale from “does not apply at all” to “fully applies.”
- The second section consisted of a total of ten knowledge questions, two for each seminar topic. Five options for an answer to each question were presented, out of which the students had to choose the correct one (multiple-choice questions). The quality criteria applied to our questions matched those of criteria for high-quality multiple-choice questions (clear question style, high degree of clarity of the answer options, no hidden clues, equal length of the answer options, questions should be able to be answered even without the answer options being visible) [19]. The knowledge test should take place 1 to 7 weeks after the last seminar to ensure long-term memory testing, but without too much time to the seminars.

One to two weeks after the end of the final seminar, each student was asked in an email to take part in the online survey and the knowledge test described above. The email explicitly stated that the survey was voluntary and anonymous. In addition, we explicitly pointed out that the potential exam questions would not be used in an official examination as part of medical school. Therefore, the students did not have to past this test. Furthermore, the students were asked not to prepare for or cheat on the test in any way, as the evaluation of the results would then lose its validity. A reminder email was sent 7–10 days after the first one.

## Results

**Fig. 1:**
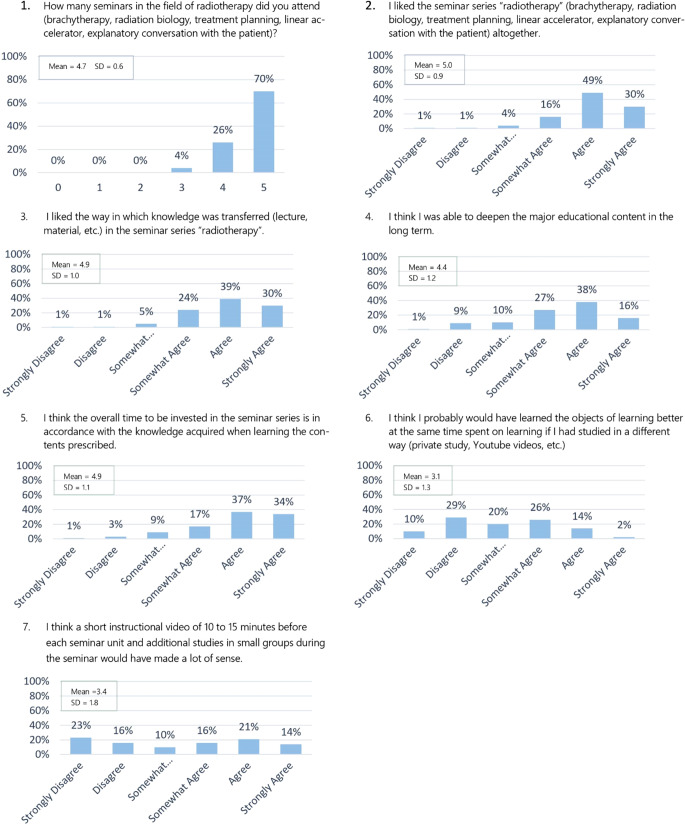
Participation, acceptance, assessment of the effectiveness in terms of learning, and on a potential use of e‑learning methods among medical students in connection with radiation oncology seminars

A total of up to 128 out of a possible 320 students took part in the survey (40%). 125 participants answered all the questions and three participants answered only the knowledge questions. Since only one miss was allowed, around 70% of the participants had attended all five, about 25% four, and only 4% three seminars. 125 participants answered all the questions referring to a personal judgement of the seminar series “radiation oncology” (Fig. 1). In general, the series was well received. About 50% of the participants liked the series, with 5 points out of 6 on the Likert scale. The average was also 5.0, with only 6% awarding 3 or fewer points (standard deviation 0.9). The result with regard to the question that referred to the judgment on the way knowledge was provided, and the required time for it, e.g., the methodical approach, was similar (standard deviation 1.0). The response to the question about the internalization of the essential content of the seminars on a long-term basis, e.g., the effectiveness in terms of learning, however, was not quite as homogenous. Although the mean was 4.4, more than 10% of the students gave a value of 3 or less. The students’ opinions on the application of alternative teaching methods or e‑learning formats, in particular on the application of the flipped classroom method, differed widely. All the numbers on the scale of 1 to 5 got about the same quantity of votes. More than 40% of the students thought that they would have internalized the essential learning content better in the long term if alternative teaching methods (self-study or instructional videos) had been applied. The distribution of votes was more homogeneous with the question on the benefits of applying e‑learning variants. Here the average was 3.4 (standard deviation 1.8).

**Fig. 2:**
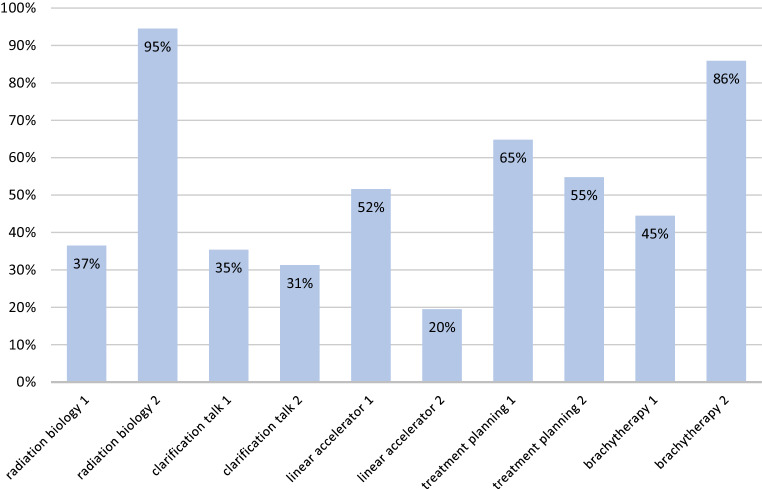
Percentage of correctly answered questions regarding radiation oncology topics among medical students

The vast majority (81%) of the students felt that they had internalized the essential learning content in the long term. This self-perception was contradictory to the results of the knowledge test, which were not satisfactory. In Germany, most tests of this type in the context of medical education require a minimum of 60% correct answers for the test to pass. Taking the performance of all the participants in the test into consideration, the percentage of correctly answered questions was 52% (Fig. 2). Students performed worst with regard to the two questions relating to the seminar “Informed consent discussion.” Only 35 and 31%, respectively, answered these questions correctly. Students performed just as poorly on the questions relating to “Target volume definition and linear accelerator.” Only 51 and 20% of the answers were correct. The best overall result was achieved concerning “Radiation biology,” with 66% correct answers on average. There was a great difference between these two questions, with 95% correct answers to one of the questions and only 37% correct answers to the other question. The questions on “Brachytherapy” achieved an average of 65%, which was almost as good as the average result for “Radiation biology.” Here too, the result of one of the two questions was above average (86%), while the other question was answered correctly by only 45% of the students. The results of the questions on “Radiation planning” (including “Radiation physics”) were in the middle of the scale. The average of correct answers was 60%, with the one question achieving 65% and the other question achieving 55%.

## Discussion

The aim of this study was to analyze the acceptance and effectiveness of traditional teaching methods in seminars concerning radiation oncology education, and to find out to what extent students might accept innovative e‑learning methods. Our study showed that traditional teaching methods are largely accepted by students, but might be less effective in terms of long-term internalization. The possible use of e‑learning methods is judged critically or desired in roughly equal parts among the students.

More than 96% of all participants in this sequence of seminars said they liked this sequence and the way in which knowledge was communicated. One explanation might be that students in general seem to prefer classes in smaller groups in comparison to traditional lectures [17]. The often practical relevance of the content and the interaction between teachers and students causes a more intimate character of these seminars. Although students mainly play a passive role in this format of teaching, they are taught about aspects of clinical routine work in a way that goes beyond regular lectures which are normally only PowerPoint presentations. The great diversity of ideas regarding application of e‑learning methods according to the flipped classroom approach makes it difficult to implement such an approach. However, it should be taken into account that there is a possibility that some students may not have had experience with the flipped classroom teaching method and were therefore unable to adequately evaluate the potential benefits of such a format. Even if the academic pattern of types of learners according to Kolb (active vs. passive, concrete vs. abstract) cannot be empirically substantiated, and one-sided attributions do not do justice to the complex personality of the individual learner, the survey may illustrate the students’ different fundamental preferences [20, 21]. About half of the students said clearly that they are basically satisfied with the current manner of learning. To them, a video as an introduction to each seminar unit would mean having to meet an additional obligation and having to spend extra time on their studies. These students obviously do not see the benefit of spending more time on the specific topic. They might also consider having a more active role in the learning process an “excessive load” of instruction. On the other hand, the other half of the students think the introduction to the current teaching session by a short video would be effective, although they are quite content with the current design of the seminars. There might be many different reasons for these two opposing opinions. For some students, the flipped classroom models might probably only get a positive rating if they had experienced this method [22]. We would suggest future analyses to elicit this issue.

In this context, the general digitalization that has conquered everyday life throughout the world might be of importance. A survey of 5000 students showed that 97% of them refer to online sources often or sometimes when studying [23]. This may lead to the conclusion that many students get convinced that this will have an additional positive effect because of the opportunity to gain a deeper understanding and remembering. However, this learning format requires more personal responsibility on the part of the students and might initially take more time [24]. Therefore it seems to be of special interest to find out to what extent the acceptance of an e‑learning format might change after its introduction, and whether there might be a clear opinion in favor of or against this format. After having participated in class and not having prepared for a test, the students were able to answer only slightly more than half of all the questions on the test correctly. In the formal exam, however, more than 80% of the answers are correct on average. The students’ self-perception regarding long-term internalization of the course content was much more positive and deviated strongly from their actual performance. This seems to indicate that traditional courses, especially seminars, are popular with students because of their connection to real clinical cases but do not stand for greater effectiveness with respect to long-term declarative knowledge transfer than other forms of teaching. We asked the students to answer the knowledge questions at a time when they were not yet preparing specifically for their exams. We asked them urgently not to specifically study for this test. The current education system means that students mainly start learning just shortly before the exam, and forget the main contents quickly afterwards (“bulimic learning”) [25]. A timespan of 1 to 7 weeks with all five seminar units seemed appropriate to us to ensure that on the one hand, the testing of the long-term memory relating to the reproduction of knowledge took place, and on the other hand, the time gap to the seminars was not too long. The results, however, reveal the importance of an additional individual repetition of the content learned in the seminar [26, 27]. Repetition could take little time if the major information given in class was introduced by a teaching video before the respective seminar, though. Thus, the additional time originally anticipated for conveying class contents and deepening knowledge would be put into perspective, since clearly less time for processing the information given and preparing for the exam would probably be needed.

The implementation of e‑learning methods, especially of instructional videos in the run-up to an in-depth seminar is complex, particularly in a cross-sectional subject such as radiation oncology. Radiation oncology combines very abstract theoretical aspects (radiation physics, radiation biology) with practically oriented clinical aspects such as explanatory conversation with the patient, patient treatment, and technical applications such as planning CT creation and target volume definition on multimodal imaging. Due to this complexity, the different aspects can be taught as a whole via e‑learning, because theoretical and practical aspects can be combined in a short time. This means, however, that the learning objectives have to be reduced to essential aspects. A short learning video of about 10 min, for example, may serve this purpose, as the structuring of the video can be optimized and the content can be selected and fully covered in advance. After the video has been produced, knowledge can be transferred outside of class without being dependent on experts or being bound to a location (e.g., in the event of illness or equipment failure), and can be accessed indefinitely and deepened at an individual pace. In this way, education can assure quality in general by utilizing instructional videos [28]. In addition, a well-made educational video should offer the opportunity to put it to use repeatedly as a self-contained unit and to understand its content without requiring much previous knowledge. Furthermore, the use of instructional videos, especially in radiation oncology, has many positive side effects. For example, brachytherapy sources may be filmed once from different perspectives in an adequate way and at high resolution, and then be presented and explained with an explanatory audio track. As a result, a presentation in class is not needed anymore, which is to be welcomed because of protection against radiation. Important aspects become redundant since they are prepared individually, and are repeated or applied in class. Repeating important aspects is substantial for a positive long-term memory performance [26, 27, 29]. Studies have also shown that active use of acquired knowledge by means of managing a specific task, for example independent conduct of an oncological informed consent discussion, is of higher value than absorbing information in a rather passive way by listening to or watching information given [27, 30–33]. Therefore, the gained time by individual preparation should be used to offer students adequate practical training, or any other way of active participation. In addition, if theoretical knowledge is connected to practical exercises, e.g., target volume definition on axial CT slices, the integration of different objectives of learning, e.g., explain target volume, recognize CT anatomy, will be reached more easily. This offers the opportunity to understand the content more deeply and remember it on a long-term basis [34]. The evidence for the effectiveness of active, cooperative, and problem-based learning is given [35]. Besides this, the chance for students to work in small groups on specific tasks leads to an increased team spirit and thus improves the students’ communication skills [34–36]. In class, the teacher adopts the position of the expert who answers any remaining questions arising from the instructional videos and may work as a moderator by asking specific questions, providing tasks, and giving supplementary information. Overall, according to several studies, flipped classroom improves student learning in terms of internalizing deep understanding of complex contents [32].

However, e‑learning or flipped classroom needs a lot of preparation time and reveals its weaknesses as soon as students come to class without being sufficiently prepared [4, 24]. For these students it will be difficult to actively participate in the discussion [37] and therefore to learn effectively.

At the Technical University of Munich, we are gaining first experience in applying modern e‑learning methods in the fields of radiation oncology. On the basis of the quality criteria mentioned above, we produced educational videos of 8–10 min for each seminar. Experts in the field of medical education and so-called eScouts (medical students who are trained to assist and advice teachers with the implementation of e‑learning formats) assisted us. The new teaching format was supposed to be launched and tested in the summer term of 2020. Unfortunately, we will have to postpone comparison of performance with students of later terms because of the decision not to have face-to-face teaching during this period due to the SARS-CoV‑2 pandemic. We were lucky to have the production of the videos completed before this decision was made, because we were therefore able to use them as teaching material in this presence-free summer term due to the COVID-19 crises. What a wonderful, though unintentional, additional argument in favor of establishing well thought out e‑learning formats.

### Limitations

The significance of the test results is limited, because due to the technical implementation of the test, we could not perform a basic quantitative analysis of the examination [38]. The analysis of item difficulty and discrimination is missing. Hence, the reliability and significance of the examination could not be determined. However, the quality criteria applied to our questions matched those of high-quality multiple-choice questions. The questions were created by the same experienced questioners as the regular examinations during the medical curriculum, and could be answered regularly with the content of the handouts for each seminar. Furthermore, it is difficult to assess the acceptance of the students regarding the implementation of e‑learning content (question 7), because it is unclear to what extent the students could imagine such a format. Both aspects can best be substantiated by a direct comparison after establishing the new e‑learning format, which will take place after the next semesters with mandatory attendance of the students.

## Conclusion

Traditional seminars enjoy a high level of acceptance among students. Effectiveness with regard to the internalization of content taught, however, should be increased. After all, the future seems to lie in the integration of e‑learning in the form of educational videos and practical seminars, because there are many advantages associated with this way of learning.
